# Vincristine in Combination Therapy of Cancer: Emerging Trends in Clinics

**DOI:** 10.3390/biology10090849

**Published:** 2021-08-31

**Authors:** Jan Škubník, Vladimíra Svobodová Pavlíčková, Tomáš Ruml, Silvie Rimpelová

**Affiliations:** Department of Biochemistry and Microbiology, University of Chemistry and Technology Prague, Technická 3, 166 28 Prague, Czech Republic; jan.skubnik@vscht.cz (J.Š.); vladimira.pavlickova@vscht.cz (V.S.P.); tomas.ruml@vscht.cz (T.R.)

**Keywords:** vinca alkaloids, antimitotics, combinatorial treatment, cyclophosphamide, doxorubicin, antibodies, procarbazine, dacarbazine, topotecan, etoposide

## Abstract

**Simple Summary:**

Vincristine is a vinca alkaloid naturally occurring in *Catharanthus roseus*. It belongs to antimitotic compounds, which arrest the cell cycle via disrupting microtubule dynamics. This property makes vincristine a useful compound in anticancer therapies, however, due to its unspecific biological action, vincristine causes severe side effects, mainly neurotoxicity. Nevertheless, at low concentrations, it is still a beneficial and widely used drug in combinatorial regimens of cancer treatment. Most commonly, it is administered with cyclophosphamide, doxorubicin, and prednisone but also with methotrexate or procarbazine, and dacarbazine. Currently, anticancer research focuses on targeted therapies and the development of antibodies specific to cancer cells. An example of such a drug combination broadly used in clinics is vincristine and rituximab, an antibody binding cancer cell surface receptor CD20. Combining vincristine with monoclonal antibodies is an emerging approach, which has been currently evaluated in clinical trials. This review article reports on the most commonly used vincristine-based drug combinations and summarizes currently running clinical trials. The number of ongoing studies shows that vincristine has its stable place in anticancer therapies and despite all its limitations, it has remained an essential part of anticancer therapies.

**Abstract:**

Treatment of blood malignancies and other cancer diseases has been mostly unfeasible, so far. Therefore, novel treatment regimens should be developed and the currently used ones should be further elaborated. A stable component in various cancer treatment regimens consists of vincristine, an antimitotic compound of natural origin. Despite its strong anticancer activity, mostly, it cannot be administered as monotherapy due to its unspecific action and severe side effects. However, vincristine is suitable for combination therapy. Multidrug treatment regimens including vincristine are standardly applied in the therapy of non-Hodgkin lymphoma and other malignancies, in which it is combined with drugs of different mechanisms of action, mainly with DNA-interacting compounds (for example cyclophosphamide), or drugs interfering with DNA synthesis (for example methotrexate). Besides, co-administration of vincristine with monoclonal antibodies has also emerged, the typical example of which is the anti-CD20 antibody rituximab. Although in some combination anticancer therapies, vincristine has been replaced with other drugs exhibiting lesser side effects, though, in most cases, it is still irreplaceable. This is strongly evidenced by the number of active clinical trials evaluating vincristine in combination cancer therapy. Therefore, in this article, we have reviewed the most common cancer treatment regimens employing vincristine and bring an overview of current trends in the clinical development of this compound.

## 1. Introduction

**Figure 1:**
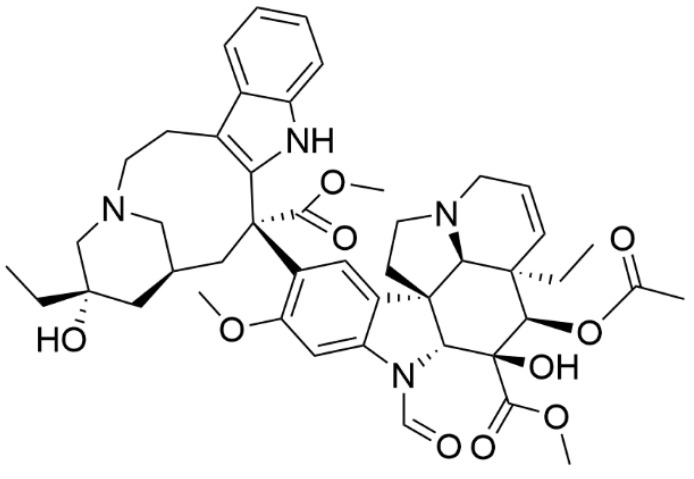
Chemical structure of vincristine.

Blood malignancies represent a less common type of cancer on a global scale, yet the number of annually diagnosed patients is not negligible. In 2020, the Global Cancer Observatory study reported almost 1.2 million confirmed cases of the three most common blood cancer types, leukemia, non-Hodgkin lymphoma, and multiple myeloma. This represents 6.2% of all reported cancer cases. More than half of these cases ended with the patient’s death, thus, blood cancers represent the cause of 6.9% of all cancer-related deaths [1]. Given these unsatisfying statistics, novel approaches to the treatment of blood cancers must be sought. The right way seems to be a combination therapy, which, in this case, often includes a naturally occurring compound vincristine (Figure 1). Vincristine is an alkaloid, which together with another anticancer compound vinblastine, occurs in Madagascar periwinkle (*Catharanthus roseus*; *Apocynaceae*). Vincristine belongs to the vinca alkaloid family, named after the *Vinca* genus, which *Catharanthus* was formerly part of. The compound was first isolated and characterized by Svoboda et al. in 1961 [2] and first approved by the US Food and Drug Administration (FDA) in 1963 for leukemia treatment under the trade name Oncovin (Eli Lilly and Company, Indianapolis, IN, USA) [3]. Currently, vincristine is mostly used in combination with other anticancer drugs and novel combinations are still being developed.

Therefore, this review aims to summarize the current trends in the development of vincristine combination therapy and brings concise evidence for the importance of vincristine in the treatment of blood cancer as well as in the treatment of other malignancies. Here, we summarize respective active clinical trials and briefly report on the mechanism of anticancer action of vincristine and the drugs used in combination with this compound.

## 2. Mechanism of Action and Side Effects of Vincristine

**Figure 2:**
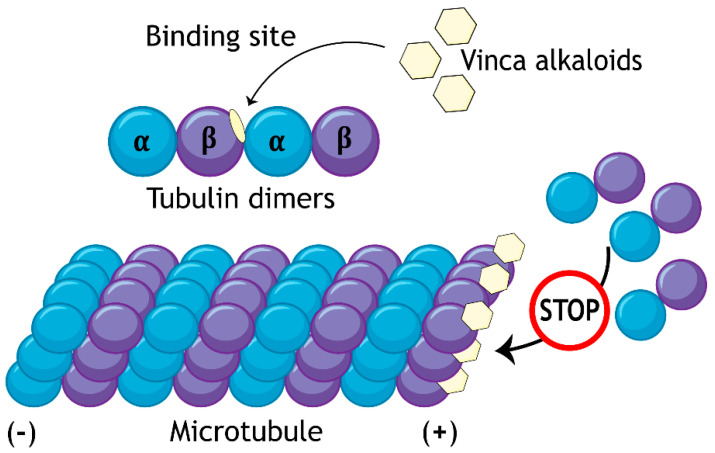
Vincristine binds the β-subunit of a tubulin dimer on the boundary with a neighboring dimer and, thus, stabilizes the microtubule dynamics by preventing binding free tubulin dimers to the microtubule fiber.

Vincristine and other vinca alkaloids belong to the group of mitotic poisons [4], particularly tubulin-binding compounds, which derive their biological properties from disrupting the function of microtubules. Microtubules are polymeric fibers composed of tubulin heterodimers. The dimers are formed by α- and β-subunits of the protein tubulin and the binding site for vincristine is located on the β-subunit at the boundary between two heterodimers (Figure 2). Vincristine and other vinca alkaloids are, thus, the only tubulin-binding agents discovered, so far, which do not strictly bind one tubulin heterodimer [5]. This important property plays a crucial role in the specific mechanism of action of vinca alkaloids. In particular, at high doses, the compounds are capable of splitting the microtubule fibers. Subsequently, the fibers join with one another always linked through the vinca alkaloid. Such irregularly ordered, often spiraled fibers are unable to fulfill their function mainly in the mitotic spindle, which is responsible for the separation of chromatids in mitosis [6]. Also, low doses of vinca alkaloids hamper this function, since the compounds bind the ends of the microtubule fibers and stabilize the microtubule dynamics [7].

The antimitotic effect of vinca alkaloids is, however, not selective to cancer cells. Therefore, naturally, there is a high risk of occurrence of severe side effects related to vincristine therapy. Most side effects caused by vincristine are identical to other antimitotic compounds since they are connected with the cell cycle disruption affecting not only cancerous cells but, unfortunately, also healthy cells. One of such affected cells can be found, for example, in the intestinal mucosa. The cell cycle arrest of the intestinal cells leads to side effects such as nausea, vomiting, or diarrhea [8]. Similarly, among other common adverse effects, there belongs hair loss, which is linked to the hair follicle cell cycle arrest [9]. Moreover, other side effects specific for vinca alkaloids exist. One of them is myelosuppression. This complicating condition is characterized by depletion of white and red blood cells, which is caused by the disrupted proliferation of progenitor cells. This problem usually occurs when chemotherapy is administered at higher doses, as the blood cells do not have enough time to repopulate between the individual doses. As a consequence of the depleted blood cell levels, sepsis, bleeding, or other complications may occur [10]. Vincristine-associated myelosuppression is a rare condition arising, for example, after overdosing [11]. Among other undesired side effects connected with vincristine therapy belong thrombocytosis [12], cellulitis (after extravasation) [13], and other unspecific conditions such as fever or skin rash [14].

However, the most severe side effect caused by vincristine therapy is neurotoxicity, which is demonstrated mainly by vincristine-induced peripheral neuropathy (VIPN). This complication dramatically impacts patients’ quality of life, it is a debilitating and very painful condition, demonstrated mainly by numbness, tingling, painful sensation in hands and feet, or muscle weakness [15]. Unfortunately, for a long time, it has not been possible to predict the probability of a VIPN outbreak in a particular patient, since the molecular basis for this effect of vincristine has been unknown. In recent years, however, some of the markers have been identified, although the significance of the published data must yet be confirmed in practice. Verma et al. employed a metabolomic approach to establish reliable predictors of VIPN. They analyzed the blood samples of 36 pediatric patients with acute lymphoblastic leukemia treated with vincristine. Out of these patients, 24 experienced high VIPN during the treatment. The samples were collected during the induction phase on days 8 and 29 and after six months of treatment. Using tandem of liquid chromatography and mass spectrometry and subsequent statistical analysis, the authors selected 2, 14, and 21 metabolites for day 8 and 29, and month 6 of treatment, respectively. Unfortunately, they were not able to determine the structure of any of the detected metabolites. On day 29, adenosine mono- and diphosphate together with *N*-acetylornithine and glycogen were identified among the metabolites. Unfortunately, no molecular pathway linked to these metabolites proves to be significantly triggered, thus, the mechanism of VIPN progression at day 29 of vincristine treatment remains unclear. Similarly, the identified metabolites after 6 months of vincristine therapy, to which belong for example oxidized glutathione or L-pipecolic acid, could not be statistically associated with the involvement of any molecular pathway in VIPN [15]. However, it has been previously described that sphingolipid levels correlate with chemotherapy-induced peripheral neuropathy [16,17,18]. Most recently, novel evidence has been published on the neuroinflammatory mechanisms of VIPN development. Starobova et al. showed that VIPN is driven by activation of the nucleotide-binding oligomerization domain-like receptors family pyrin domain-containing 3-inflammasome and subsequent release of interleukin-1β from macrophages. These neuroinflammatory mechanisms lead to mechanical allodynia and gait disturbances. In addition, the authors showed that VIPN progression in vincristine-treated medulloblastoma mice models is prevented by the antagonist of the interleukin 1 receptor (IL-1) called anakinra (Kineret), suggesting that this substance might be a suitable agent for combinational therapy with vincristine [19]. Combination therapy, in general, is a massively applied approach in vincristine treatment enabling mainly reduction of vincristine doses. In the next chapters, the most common combination therapies and currently the most clinically tested approaches will be reviewed.

## 3. Combinations of Vincristine with Cyclophosphamide, Doxorubicin, and Prednisone

**Figure 3:**
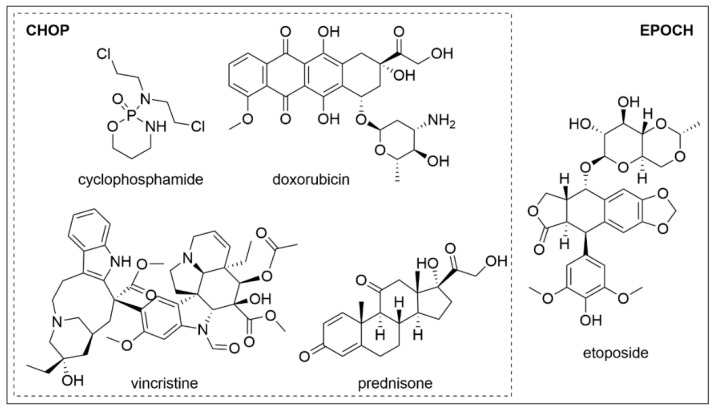
Chemical structures of cyclophosphamide, doxorubicin, vincristine, and prednisone (together forming the CHOP regimen) and etoposide (with CHOP forming the EPOCH regimen).

Probably the most well-known drug combination including vincristine is so-called CHOP, which is a combination anticancer chemotherapy using cyclophosphamide (Cytoxan^®^, developed by Asta-Werke Aktiengesellschaft Chemische Fabrik, Brackwede, Germany), doxorubicin hydrochloride (Adriamycin^®^, Pfizer^®^, New York, NY, USA), vincristine sulfate (Oncovin^®^, Eli Lilly and Company, Indianapolis, IN, USA), and prednisone or prednisolone (Meticorten^®^ or Delta-Cortef^®^ respectively, introduced to the market by the Upjohn Company, Kalamazoo, MI, USA). The combination of the listed drugs (Figure 3) has long been considered the standard regimen to treat non-Hodgkin lymphoma [20]. The cytotoxic action of this combination is based mainly on targeting DNA. Cyclophosphamide, which was approved in 1959, is a widely used alkylating agent effectively inducing DNA cross-linking [21]. Next, doxorubicin intercalates into the DNA and binds proteins involved in DNA replication and transcription, which results in inhibition of DNA, RNA, and protein synthesis followed by cell death triggering [22]. Thanks to this action, doxorubicin was approved by the FDA in 1974 for the treatment of soft tissue sarcoma [23]. Besides the aforementioned cytotoxic drugs, corticosteroids prednisone and prednisolone are administered in the CHOP regimen to decrease cancer-related pain based on their anti-inflammatory properties [24].

Currently, CHOP is used more often in combination with rituximab (R-CHOP). Rituximab (Rituxan^®^, developed by Biogen, Cambridge, MA, USA) is a chimeric anti-CD20 IgG1 monoclonal antibody. Its target, the CD20 molecule, is a cell surface protein that plays a key role in the development and differentiation of B cells [25]. Rituximab alone was approved by the FDA in 1997 for the treatment of indolent forms of B cell non-Hodgkin lymphoma. Rituximab combination with CHOP was approved nine years later [26]. The addition of rituximab to the CHOP regimen potently increases the patients’ outcome, however, still about one-third of patients’ relapses [27]. Thus, this combination is currently evaluated with additional drugs in clinical trials for the treatment of different types of cancer (Table 1). One of the standardly used drugs in combination with CHOP or R-CHOP is etoposide (Vepesid^®^, Bristol-Myers Squibb, New York, NY, USA). This drug was approved by the FDA in 1983 and it acts via interacting with topoisomerase II, in particular with ligation activity of this important DNA topology-ensuring enzyme. Thereby, an increased amount of cleaved DNA occurs in cells after etoposide treatment [28]. The combination of etoposide with R-CHOP is termed R-CHOPE or more commonly EPOCH-R, which will be also used in this article. It appears to be effective in some specific types of aggressive B-cell lymphomas, which respond poorly to R-CHOP alone, such as lymphomas connected with the rearrangement of the *c-myc* gene. As shown in a prospective, multicenter, single-arm phase 2 study, dose-adjusted EPOCH-R produces durable remission in patients bearing the *c-myc* rearrangement [29]. Similar to the lymphoma with *c-myc* rearrangement, EPOCH-R might be useful in Burkitt lymphoma treatment. This aggressive type of B cell lymphoma, common in children, is currently treated with intensive combination regimens including CHOP and methotrexate [30]. Unfortunately, these regimens, which were developed primarily for children, cannot be used in adult patients, mainly in those with comorbidities (for example human immunodeficiency virus), since combinations with methotrexate are often toxic. However, in these patients, EPOCH-R seems to be effective, as confirmed in a multicenter study [31]. In addition to the aforementioned lymphoma types, EPOCH-R could find its use also in the treatment of primary mediastinal large B cell lymphoma. The efficacies of EPOCH-R and R-CHOP against this cancer are comparable, however, in clinical practice, R-CHOP is standardly combined with radiotherapy in this case. In the multicenter analysis performed by Shah et al. EPOCH-R is greatly effective even without radiotherapy, which would be a great advantage of such treatment [32].

EPOCH-R can be further combined with a variety of drugs. One of such is ixazomib (Ninlaro^®^, Takeda Pharmaceutical Company Limited, Tokyo, Japan), an FDA-approved proteasome inhibitor, which can downregulate *MYC*. This may be useful in the treatment of certain lymphomas, as discussed above. After all, the efficacy of ixazomib was shown in clinical trials [58]. The data from phase I/II clinical study of EPOCH-R plus ixazomib in adult patients with *MYC*-aberrant lymphoid malignancies show good tolerability and safety of such combination. Unfortunately, 45% of the patients did not finish the induction phase with planned doses of ixazomib. In addition, only 55% of patients received maintenance therapy and only less than 35% of them finished the treatment without discontinuation or dose reduction. The reasons for such changes during the treatment included mainly peripheral neuropathy, patient preference, and progressive disease. However, the patients, who finished the treatment, showed good outcomes, mainly a strong tumor reduction [58]. EPOCH-R plus ixazomib might be a useful drug combination, nevertheless, further studies are necessary. Besides ixazomib, another proteasome inhibitor has been tested in clinical trials with R-CHOP, EPOCH, and EPOCH-R. Bortezomib (Velcade^®^, Takeda Pharmaceutical Company Limited, Tokyo, Japan), a substance with a broad spectrum of biological actions, has been tested in the treatment of lymphoma, in which it acts through upregulation of the gene encoding phorbol-12-myristate-13-acetate-induced protein 1 (*PMAIP1*) mediated by oxidative/endoplasmic reticulum stress- (also known as *NOXA*; a gene encoding phorbol-12-myristate-13-acetate-induced protein 1) and subsequent induction of apoptosis. In a randomized phase II study in patients with non–germinal center B-cell–like diffuse large B-cell lymphoma, the addition of bortezomib to R-CHOP did not show significant improvement in patients’ outcomes [59]. A phase III clinical trial confirmed that the exchange of vincristine for bortezomib in the R-CHOP regimen significantly improves survival rates in patients suffering from mantle cell lymphoma [60]. This raises the question of whether vincristine should not be omitted or at least exchanged for other drugs in the combinatorial regimens. However, the number of currently running clinical trials with vincristine shows rather the opposite (Table 2). Since CHOP and its modifications are not the only combinational regimens with vincristine, which are standardly used in cancer treatment.

## 4. Combination of Vincristine with Methotrexate

Besides various combinations based on CHOP, vincristine has commonly been combined with methotrexate (Trexall^®^, Otrexup^®^, or Rasuvo^®^, originally developed by Bound Brook researchers), which is a selective inhibitor of the dihydrofolate reductase, an enzyme vital for the thymine nucleotide synthesis [65]. Although the vincristine/methotrexate combination has been tested and used in clinics since the 1960s, its benefits are still discussed. Initial trials evaluated the combination of these two drugs with cyclophosphamide and 5-fluorouracil, which also blocks the synthesis of thymidine. The trials presented positive outcomes in various solid tumors, however, in one of the first studies published on this topic, only a half of the involved patients responded objectively to the treatment and almost all patients were affected by the toxicity of the drug combination [66]. The latter combination of four drugs was further extended with prednisone and evaluated in a pilot clinical study on patients with different solid tumors. This combination seems to be more effective and well-tolerated since minimal or no toxicity was observed in 47% of tested patients and 51% had mild side effects. The best response was observed in ovarian cancer patients, 45% of which responded at least partially [67]. The rationale for the beneficial administration of vincristine with methotrexate is based on the fact that vincristine potently increases the uptake of methotrexate into cells and makes the methotrexate treatment more effective [68].

Despite promising results from early studies, further published data are inconsistent. In 1979, Mulder and Putten reported no significant benefit of vincristine administration on methotrexate toxicity in solid tumor mice models. In leukemia mice models, they also showed that the strategy to administer vincristine before methotrexate does not lead to enhanced effectivity in comparison to the simultaneous administration of the two drugs. Importantly, the authors found out that pretreatment with vincristine leads to methotrexate-induced weight loss, and in most of the mice it leads to early toxic death [69]. A different situation, however, occurs when methotrexate is administered before vincristine. In this reversed layout, the synergistic action of the two compounds occurs both in vitro and in vivo increasing mainly at a prolonged interval between administration of both drugs [70,71]. The benefits of the use of vincristine in the regimens containing methotrexate have been discussed also more recently. Freeman et al. published a retrospective review of 29 patients receiving high-dose methotrexate induction therapy in combination with rituximab, procarbazine (Matulane^®^, Leadiant Biosciences, Pomezia, Italy; alkylating agent), and, for half of the patients, vincristine [72]. This combination belongs to the recommended regimens for the treatment of primary central nervous system (CNS) lymphoma (PCNSL). However, vincristine is a weak point of the regimen due to dose-limiting neuropathies. Freeman et al. found out that the outcomes were similar regardless of vincristine inclusion in the regimen. In the group receiving vincristine, however, more severe adverse effects occurred. The authors, thus, propose larger studies to evaluate the efficacy of vincristine in the treatment of PCNSL.

Currently, methotrexate and vincristine are being tested in larger combinations with other drugs. Promising results were obtained in phase II clinical trial in patients with newly diagnosed intravascular large B-cell lymphoma, who received R-CHOP with high-dose methotrexate. This combination was further extended with intrathecal chemotherapy containing methotrexate, cytarabine (Cytosar-U^®^, first marketed by Upjohn, Kalamazoo, MI, USA; DNA synthesis inhibitor), and prednisolone. The regimen was proven safe, with minimum severe side effects, and is, thus, recommended for further phases of the clinical trials on lymphoma patients with CNS involvement [73]. Besides vincristine and methotrexate, another effective drug combination includes etoposide, cyclophosphamide, and dactinomycin (also actinomycin D, Comsegen^®^, initially marketed by Merck & Co., Kenilworth, IL, USA; inhibitor of transcription). This regimen, commonly termed EMA/CO, is standardly used for the treatment of gestational trophoblastic cancer. Statistical analyses report excellent prognosis and survival rates in patients treated with EMA/CO, mainly after induction therapy with etoposide and cisplatin [74,75,76]. However, the most recent study on this combination again points to the risks of the vincristine treatment, since when compared with methotrexate, etoposide, dactinomycin (together abbreviated as EMA), the benefits were similar, but EMA/CO-treated patients suffered more often from neutropenia and peripheral neuropathy [77]. Thus, the real contribution of the latter combination must be confirmed or disproved in further trials. In general, however, wide regimens containing sundry drugs together are one of the most common solutions in current cancer medicine and must still be developed to achieve the best response [78].

## 5. Combination of Vincristine with Procarbazine or Dacarbazine

**Figure 4:**
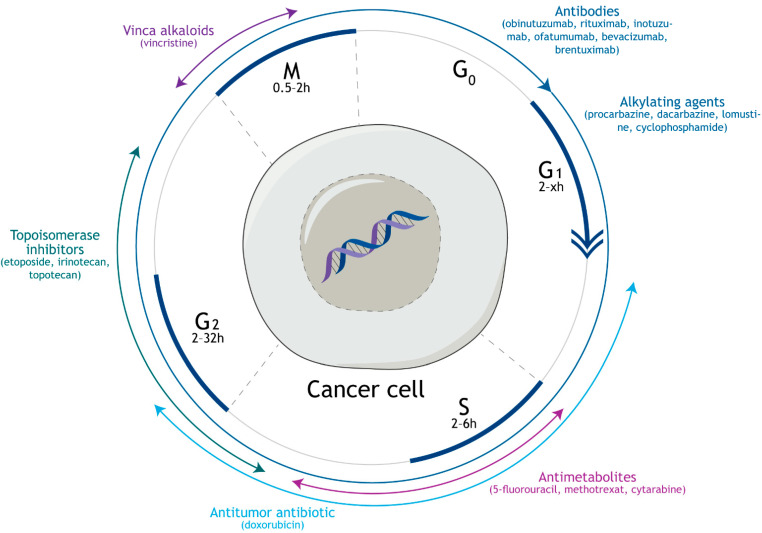
Phases of the cell cycle, which are affected by vincristine, and the compounds, which are used in combination with vincristine in cancer treatment.

Vincristine is a part of a very broad spectrum of anticancer combinations consisting of drugs with various mechanisms of action (Figure 4). Many of these combinations contain procarbazine and dacarbazine (DTIC-Dome^®^, Bayer, Leverkusen, Germany), which are low-molecular-weight compounds with anticancer activity acting as methylating agents. They can methylate guanine at the *O*-6 position and, therefore, impede the process of DNA replication during cell division. Combinations of these agents with vincristine are currently commonly used in clinical practice for the treatment of various tumor types. A very well-established anticancer regimen, with the abbreviation PCV, contains procarbazine, vincristine, and lomustine (Gleostine^®^, manufactured by NextSource Pharmaceuticals; a nitrosourea derivative acting as alkylating agent [79]). PCV has been used to treat mainly malignancies of the CNS [80], such as anaplastic oligodendrogliomas [81], high and low-grade gliomas (LGG) [82], or anaplastic astrocytomas [83]. Best treatment results are achieved when PCV is applied together with radiotherapy. Recently, results of a phase III study have been published, confirming the benefit of PCV administration in patients with LGG treated with radiotherapy. The study aimed to compare the efficacy of the combined treatment on different types of LGG, particularly those with or without a mutation in the *IDH1/2* gene encoding nicotinamide adenine dinucleotide phosphate-dependent isocitrate dehydrogenases [84]. This mutation was identified before as a weak point for the PCV treatment, as Cairncross et al. published their results from a clinical trial on oligodendroglioma patients and reported longer survival rates by patients with mutated *IDH* [85]. Based on the aforementioned recent study, however, it is not so simple to assess the PCV benefits. The addition of this regimen to radiotherapy certainly improves outcomes from LGG treatment regardless of *IDH* mutation status. Patients with *IDH* mutation live longer after the combined treatment, though they need stronger regimens and, generally, the type of LGG with *IDH* mutation is more aggressive [84].

Vincristine and procarbazine have formerly been used as a standard treatment of Hodgkin´s lymphoma in a combination with prednisone and mechlorethamine, which is nitrogen mustard belonging to DNA-interacting agents able to alkylate and crosslink DNA. This combination of the four drugs abbreviated as MOPP is currently used only rarely since alkylating agents are connected with a high risk of induced secondary malignancies. The adverse effects of MOPP were discovered soon after its introduction into the clinics and comprise mainly leukemia development [86,87]. So far, no evidence has been reported on vincristine being a secondary cancer inducer. These effects are most probably connected only to the mechanism of action of alkylating agents [88]. Despite that, DNA-interacting compounds are still being used in cancer therapy and are commonly combined with vincristine.

One of such combinations is composed of cyclophosphamide, vincristine, and dacarbazine (together abbreviated as CVD). Mostly, it has been used in the treatment of malignant pheochromocytoma. Although the first evidence of the efficacy of CVD was published in 1988 [89] and brought great hope for pheochromocytoma patients, though, the actual benefit of this combination has not yet been proved. The published trials reported partial patient responses, however, the percentage of the responding patients was rather low (less than 50%). Unfortunately, it was also difficult to distinguish the effect of the treatment from the natural progress of the disease [90,91]. In addition, a recent study on CVD in the treatment of pheochromocytoma reported very inconclusive results. Only one-third of patients responded to the treatment, however, a slight biochemical improvement was observed also in the non-responding group. The authors, therefore, suggested some usefulness of CVD in controlling tumor progression, but further studies need to be done [92]. The usefulness of CVD in stabilizing the tumor progression was proved also in advanced medullary thyroid carcinoma [93]. Given all the published studies, it is clear that the efficacy of CVD is strongly dependent on the particular patient and his condition. Without knowing the exact mechanism of action or at least without a large clinical study and statistical analysis, we will not be able to use the CVD combination effectively. Nevertheless, other combinations of vincristine and dacarbazine are more successful in clinics.

In addition, cyclophosphamide, vincristine, and dacarbazine were also combined with bleomycin (Blenoxane^®^, Bristol-Myers Squibb, New York, NY, USA), which is a non-heme iron protein, acting through induction of DNA strand breaks. A very common regimen consists of doxorubicin, bleomycin, vincristine, and dacarbazine, abbreviated as ABVD. This combination, only with procarbazine instead of dacarbazine, has been further extended with etoposide, cyclophosphamide, and prednisone, together termed as BEACOPP. Both combinations are commonly indicated as the first-line treatment of non-Hodgkin lymphoma and for some indications of leukemia and other blood malignancies. The extended combination seems to be slightly more effective than ABVD in leukemia treatment, as presented in a pooled analysis of four independent clinical trials [94]. In non-Hodgkin lymphoma, BEACOPP is more effective than ABVD in progression-free survival but not in overall survival [95]. The statistical data confirm that the strategy of using wider drug combinations, especially when vincristine is included, represents the right way in modern cancer treatment. At least before highly targeted therapies will be fully tested and implemented instead of combination therapies.

A current trend in clinical trials is even to further combine ABVD or BEACOPP with other drugs, for example just with the specific tumor targeting molecules. One of the most recently published studies evaluated the benefits of the addition of brentuximab vedotin (Adcetris^®^, Takeda Pharmaceutical Company Limited, Tokyo, Japan; Seagen, Bothell, WA, USA), which consists of a monoclonal antibody against the membrane protein CD30 and an antimitotic compound monomethyl auristatin B. In phase III of a clinical trial on patients suffering from Hodgkin lymphoma, a combination of ABVD with brentuximab vedotin was more effective than ABVD only, with a 4.9% lower risk of death, progression, or incomplete response [96]. Similar results were obtained in a parallel phase II study, conducted by the German Hodgkin Study Group. This study, however, omitted to evaluate vincristine as part of the therapeutic regimen. The most efficient variant of BEACOPP treatment consisted of brentuximab vedotin, etoposide, doxorubicin, cyclophosphamide, dacarbazine, and dexamethasone (originally approved under trade name Decaspray^®^, Merck & Co., Kenilworth, IL, USA) [97]. In general, brentuximab vedotin seems to gradually replace vincristine in the multidrug anticancer combinations for Hodgkin lymphoma treatment [98]. However, the cost-effectiveness of such treatment is arguable, since the improvement of patient outcomes is not so high to balance the high cost of the antibody [99]. This aspect cannot be omitted in considering the overall benefit of brentuximab vedotin in addition to both aforementioned combinations. ABVD or BEACOPP regimens alone are very effective against blood cancer malignancies, yet, their further development is still needed. Evidence suggests that these regimens could negatively impact the fertility of both male and female patients [100,101]. In addition, Ramos et al. reported that the ABVD regimen induces genomic chaos after one year of the treatment. The chromosomal abnormalities caused by the treatment may subsequently lead to secondary malignancies [102]. Although the latter two examples of adverse effects of vincristine combinatorial treatment in Hodgkin lymphoma patients lack deeper evidence, it suggests that further study of the discussed combination and perhaps also further manipulations with their composition are needed.

## 6. Other Vincristine Combination Therapies

Modern anticancer research is practically unlimited in combining different drugs to achieve the best possible effectiveness. Also in the case of vincristine, there are much more options besides the aforementioned most commonly used ones. A common drug, administered often in wider combinations, is a DNA interacting compound carboplatin (Paraplatin^®^, Bristol-Myers Squibb, New York, NY, USA). The carboplatin vincristine regimen is used as first-line chemotherapy for childhood LGG [103]. This regimen has been further extended with other drugs. Whereas the addition of etoposide does not increase the efficacy of the regimen in LGG [103], the combination with alkylating agent temozolomide (Temodar^®^, Merck & Co., Kenilworth, IL, USA) seems to be an effective option for this indication [104]. Besides etoposide and temozolomide, vincristine and carboplatin were tested also in combination with topotecan (Hycamtin^®^, GlaxoSmithKline, London, UK), which is a topoisomerase inhibitor. The clinical trial of this three-drug combination has been withdrawn for the long-term risk of secondary malignancies, but the combination of vincristine with topotecan alone has continued to be further studied [105]. This combination is most suitable for the treatment of retinoblastoma. In patients with this diagnosis, it enables chemoreduction and is well effective already after two cycles [106]. In addition to retinoblastoma, also neuroblastoma might be potentially treatable with vincristine and topotecan co-administered with doxorubicin. According to the pivotal study, this combination is safe and improves the response rate in patients receiving cisplatin, vincristine, etoposide, cyclophosphamide, and carboplatin as an induction therapy [107]. Also, cyclophosphamide is a suitable drug to be combined with vincristine and topotecan. In Ewing sarcoma, such a combination seems to be profitable, however, the statistical analysis of a larger group of patients is needed to confirm the efficacy over the standard regimen, which now consists of vincristine, doxorubicin, cyclophosphamide, ifosfamide (Ifex^®^, Baxter International, Deerfield, FL, USA, DNA synthesis inhibitor), and etoposide [108]. The use of vincristine can be well demonstrated by the example of Ewing’s sarcoma. Similar to various other indications, as mentioned before, vincristine is used as the standard therapy, but at the same time, it is a part of the experimental regimens. Thus, it so far plays an irreplaceable role in the current cancer medicine.

A very important role in the current anticancer treatment is emerging also for cancer-specific monoclonal antibodies. As mentioned before, some of them are already well-established in combination with vincristine, mainly rituximab, which is administered with CHOP and EPOCH regimens. Among antibodies recently tested with vincristine is also bevacizumab (Avastin^®^, Genentech, South San Francisco, CA, USA), a monoclonal antibody against vascular endothelial growth factor-A. Especially the combination of vincristine, bevacizumab, irinotecan (Camptosar^®^, Pfizer, New York, NY, USA; inhibitor of topoisomerase I), and temozolomide could be useful in the treatment of childhood cancers [109,110,111,112,113]. Bevacizumab was, similarly to rituximab, evaluated also in combination with CHOP in phase II clinical trial in patients suffering from peripheral T-cell lymphoma. However, this combination is not beneficial even significant toxicities have been observed [114]. Nevertheless, CHOP has also been combined with other antibodies, such as obinutuzumab (Gazyva^®^, Genentech, South San Francisco, CA, USA), a monoclonal antibody against membrane protein CD20. The regimen of CHOP plus obinutuzumab reached phase III clinical trial in patients with diffuse large B cell lymphoma. Its results, however, did not prove a significant benefit over the R-CHOP combination [115]. In addition to obinutuzumab, vincristine has been tested also with other anti-CD20 antibodies, including ublituximab (LFB Group, Alès, France) [116], mosunetuzumab (Genentech, South San Francisco, CA, USA) [117] targeting also cluster of differentiation 3 (CD3), glofitamab (Genentech, South San Francisco, CA, USA) [118], or ofatumumab (Arzerra^®^, Novartis, Basel, Switzerland) [119], which has been proven safe and prolonged progression-free survival in elderly patients suffering from diffuse large B-cell lymphoma in phase II clinical trial in combination with CHOP [120]. CD molecules occur very specifically in cancer cells and are, thus, a popular target for therapy. Besides CD20 antibodies, an anti-CD2 antibody siplizumab (BioInvent, Lund, Sweden) reached clinical trials in combination with EPOCH-R in aggressive peripheral T-cell lymphomas [121]. Unfortunately, given the so far reported data, this regimen is not able to prolong progression-free survival [122]. A possibly more efficient option could be the involvement of inotuzumab ozogamicin (Besponza^®^, Pfizer, New York, NY, USA), a combination of anti-CD22 antibody and a cytotoxic agent from a calicheamicin family. A combination of these drugs with cyclophosphamide, dexamethasone, vincristine, methotrexate, and cytarabine was evaluated in a phase II clinical trial on older patients with Philadelphia chromosome-negative acute lymphoblastic leukemia. The results from this trial show this combination to be safe and active as the first-line treatment for the indicated condition [123,124]. Among other antibodies targeting a CD molecule tested with any drug combination including vincristine are anti-CD19 Tafasitamab (Monjuvi^®^, MorphoSys, Planegg, Germany) [125,126,127] and blinatumomab (Blincyto^®^, Amgen, Thousand Oaks, CA, USA) [128,129,130,131,132,133,134], which targets also CD3; anti CD30 brentuximab (Adcetris^®^, Takeda Oncology, Cambridge, MA, USA) [135,136,137,138]; anti CD38 daratumumab (Darzalex^®^, Janssen Biotech, Horsham, PA, USA) [139,140] and isatuximab (Sarclisa^®^, Sanofi, Paris, France) [141]; anti CD52 alemtuzumab (Campath^®^, Sanofi, Paris, France) [142,143,144] and anti CD79B polatuzumab (Polivy^®^, Genentech, South San Francisco, CA, USA) [145,146]. However, CD molecules are not the only ones, which can be targeted by antibodies during cancer therapy. A very commonly used target for antibodies has been also programmed cell death-ligand 1 (PD-L1), which plays a role in curtailing the response of activated T cells at sites of infection preventing the development of autoimmunity [147]. Sundry anti-PD-L1 antibodies have been clinically evaluated in combination with vincristine, namely atezolizumab (Tecentriq^®^, Roche, Basel, Switzerland) [148,149], nivolumab (Opdiva^®^, Bristol-Myers Squibb, New York, NY, USA) [150,151,152], toripalimab (Tuoyi^®^, Junshi Biosciences, Shanghai, China) [153], pembrolizumab (Keytruda^®^, Merck & Co., Kenilworth, IL, USA) [153,154], camrelizumab (AiRuiKa^®^, Jiangsu Hengrui Medicine, Lianyungang, China) [155], durvalumab(Imfinzi^®^, MedImmune, Gaithersburg, MD, USA) [156], sintilimab (Tyvyt^®^, Eli Lilly and Company, Indianapolis, IN, USA) [157,158], and avelumab (Bavencio^®^, EMD Serono, Rockland, USA; Pfizer, New York, NY, USA) [159]. Furthermore, vincristine has been clinically tested also with an antibody raised against ganglioside G2, dinutuximab (Unituxin^®^, United Therapeutics, Silver Spring, MD, USA) [160], and with an antibody against the type 1 insulin-like growth factor receptor, ganitumab (Amgen, Thousand Oaks, CA, USA) [161]. The big number of studied combinations of vincristine with antibodies against various targets prove that this strategy is a vivid option in cancer combating. Further studies are currently in progress, summarized in Table 3.

In addition to all the aforementioned clinically developing drug combinations including vincristine, this compound has currently been combined with sundry not yet clinically evaluated compounds. These compounds mostly comprise inhibitors of P-glycoprotein (P-gp) efflux pumps, which can sensitize resistant cells to the correspondent drugs. Vincristine belongs to the drugs, which are efficiently transported from cells via P-gp, and in some cancer cell types overexpressing this efflux pump, leads to strong resistance to vincristine treatment [175]. The group of Chen et al. presented a series of seco-based compounds derived from a previously reported compound praeruptorin A, which potently reverses P-gp-based multidrug resistance. Out of 25 synthesized derivatives, two showed extremely beneficial actions when co-administered with vincristine. The half-maximal inhibitory concentration (IC_50_) of vincristine decreased dramatically from 666.3 nM concentration when administered alone to 0.84 and 0.53 nM concentration after co-administration with the two most potent seco-based derivatives (evaluated in cervical adenocarcinoma KB-V cell line after 48 h of incubation). The novel seco-based derivatives were active only in P-gp overexpressing cells, which points to their mechanism of action as P-gp inhibitors [176]. The exact mechanism of their action must yet be elucidated, however, it is probably a direct inhibition by binding to the efflux pump, since the compounds do not decrease the expression levels of P-gp [177]. P-gp targeting is a successful approach in improving vincristine anticancer activity. Li et al. recently developed 5-phenylfuran derivatives, one of which was proven effective in terms of sensitizing resistant breast cancer cells MCF-7/ADR overexpressing P-gp to vincristine treatment. While IC_50_ of vincristine alone after 48 h in these cells is 10.05 µM, when given in combination with the 5-phenylfuran derivative, the IC_50_ is equal to 0.16 µM concentration [178]. Another example of a P-gp inhibitor, synergizing with vincristine, has been presented by Syed et al., who computationally designed, synthesized, and evaluated two piperine analogs, one of which was proven to potentiate vincristine anticancer activity. This compound was able to reduce vincristine IC_50_ in resistant KB cells 24-fold while being inactive in non-resistant cells [175]. Not only synthetic products but also compounds from natural sources have anti-P-gp activity and were tested in combination with vincristine. Teng et al. reported 5-hydroxy-7,8-dimethoxyflavanone from *Fissistigma cupreonitens* to strongly bind P-gp and increase the cytotoxic effect of vincristine. In P-gp expressing cells, ABCB1/Flp-In^™^-293 increased the IC_50_ of vincristine after 72 h of co-administration with the novel P-gp inhibitor 23-fold in comparison to the non-P-gp-expressing analog [179].

All the mentioned studies show a great potential of combining vincristine with P-gp inhibitors and hopefully, these compounds will soon emerge in preclinical and clinical evaluation. However, targeting P-gp is not the only approach to sensitize cells for better vincristine performance. Diouf et al. discovered that by decreasing the expression of non-histone protein 2-like protein 1 (NHP2L1), it is possible to reduce the adverse neurotoxic effects of vincristine and, thereby, to enable more effective treatment. They identified tazarotene, quetiapine fumarate, and dipyridamole to be potent inhibitors of NHP2L1 and proved them to increase intracellular levels of vincristine (most probably via P-gp inhibition) and protect neurons from vincristine neurotoxicity in mice bearing human leukemia cells [180]. Another group of compounds, which could be beneficial when administered with vincristine are autophagy inhibitors. Autophagy is a well-characterized process and its role in cancer development has been extensively studied. Some chemotherapeutics such as vincristine can trigger autophagy. This would seem beneficial as autophagy is a type of cell death; however, in cancer therapy, this process is a double-edged sword. Under stressful conditions, for example, during chemotherapy, cells can use autophagy to increase their survival rate and, thus, decrease the therapy outcome. The phenomenon of autophagy in cancer treatment has been recently reviewed in [181]. Administration of autophagy inhibitors together with anticancer compounds, which trigger autophagy, is a modern effective approach for the treatment of resistant types of cancer. Also, vincristine can be combined with autophagy inhibitors. Xia et al. reported a strategy on overexpressing the maternally expressed gene 3 (*MEG3*) encoding a long non-coding RNA (lncRNA) acting as a tumor suppressor. The expression of this RNA in vincristine-treated lung cancer cells (A549) is decreased and autophagy is triggered. However, when transfected with *MEG3* plasmid DNA, the levels of autophagy proteins LC3-I and LC3-II (microtubule-associated proteins 1A/1B light chain 3B) decreased significantly and the cells were sensitized for vincristine treatment, which was then more effective than in non-transfected cells [182]. Another study on lnc-RNAs and autophagy has been published by Yao et al., who sensitized cells for vincristine treatment by silencing X-inactive specific transcript (XIST), which is an lncRNA acting as a tumor growth accelerator. In retinoblastoma cells (WERI-Rb1 and Y79), autophagy was decreased more than by twofold after 48 h of cell transfection with plasmid DNA encoding short-interfering RNAs against XIST. Additionally, in mice xenograft models of retinoblastoma cells with silenced XIST, vincristine treatment (1 mg·kg^−1^, every 3 days for 22 days) resulted in a potent decrease in the tumor volume and weight (more than twofold in comparison to XIST-expressing counterparts) [183]. Targeting lnc-RNAs both in terms of their up-and downregulation seems to be a vital strategy against vincristine-resistant cells. Besides lnc-RNAs, the use of small-molecular autophagy inhibitors can also potentiate vincristine anticancer activity. In nephroblastoma cells (G401), a commonly used autophagy inhibitor chloroquine (10 µM) increased the cytotoxicity of vincristine by 15%, when vincristine was administered at low concentrations (1–2 nM) for 24 h [184]. Similarly, a novel autophagy inhibitor *N*-(cyclohexylmethyl)-5(((cyclohexylmethyl)amino)methyl)-2-((4-(trifluoromethyl)benzyl)oxy)benzamide reported by Shan et al. synergizes with vincristine to enhance cytotoxicity in resistant esophageal cancer cells (Eca109/VCR) [185]. In general, inhibition of autophagy is beneficial to eliminate cancer cell resistance to vincristine. The latter studies show a promising future of vincristine since many novel compounds are still being discovered and evaluated in combination with this vinca alkaloid to improve its therapeutic outcome.

## 7. Conclusions

Vincristine is a vinca alkaloid, which derives its anticancer activity from destabilizing microtubule fibers and arresting the cell cycle in the G2/M phase. Given its low cancer cell selectivity, this drug commonly causes undesired side effects, the most serious of which is peripheral neuropathy. This is why vincristine should be used at lower concentrations and, therefore, is suitable rather for combination therapy than monotherapy. So far, vincristine has been used in multiple anticancer regimens, both as standard therapy or clinical research. The most commonly known drug combinations including vincristine are CHOP and its variants. These regimens are standardly used in the treatment of non-Hodgkin lymphoma and other blood malignancies. Despite the fact, still, the response of the patients is not fully predictable and the success of CHOP-based therapy depends on complex conditions of the particular patients. Therefore, such regimens still undergo active development. For example, the addition of rituximab has dramatically improved the efficacy of CHOP-based cancer treatment, so it is now standardly administered as part of the treatment regimens. Their further improvement with etoposide addition enabled even to omit radiotherapy, which is a standard part of the CHOP-based procedure.

A promising drug combination with vincristine, different from CHOP regimens, includes methotrexate. The clinical data for such combination are, however, contradictory. Although recent studies that tested both drugs in combination with other compounds (EMA/CO regimen), showed good patient outcomes and tolerability, one study reported neuropathy and neutropenia in the vincristine-containing group and similar activity of the regimen without vincristine. An important question, thus, is whether vincristine still should be included in anticancer regimens if such side effects are still a threat. The amount of currently ongoing clinical trials with this antimitotic compound clearly shows that vincristine has its place in modern anticancer approaches. At the moment, 156 active trials are registered and 219 are in the preparation phases. Thus, not only CHOP and methotrexate-based regimens, but also combination therapies with procarbazine and dacarbazine, carboplatin, or topotecan have been extensively studied and some of them are already standardly used in clinics. One of the most modern approaches in anticancer treatment, the use of cancer-specific antibodies, is also combined with vincristine regimens. Anti-CD20 antibody rituximab is clinically administered with CHOP and EPOCH, other antibodies in combination with vincristine are currently in development.

The so far reported results of the realized clinical trials bring strong evidence of the high usefulness of vincristine-based drug combinations for the treatment of various types of cancer. It is surprising that despite numerous side effects connected with vincristine-based treatment, this compound is still irreplaceable in clinics. Is that because none of all the tested compounds so far is more effective? Or is just vincristine effective enough that it is not necessary to seek novel compounds, which would once replace vincristine? Rather the first question is right. Although there are a lot of anticancer drugs and far more drug candidates currently in clinics or clinical evaluation, none of them is that effective to replace vincristine. Mainly in pediatric cancer plays vincristine the utmost important role. It could be seen in 2019 as there was a great shortage of vincristine supplies. Vincristine is the first-choice treatment for pediatric acute lymphoblastic leukemia, the most common cancer among children. Also due to the great effort of many children’s cancer organizations, such as the American Childhood Cancer Organization, one of the vincristine-manufacturers decided to restore the stopped production of the drug [186]. Even now, according to FDA, there is a shortage of vincristine in the U.S. [187]. There are no indications that vincristine could soon be replaced by another drug, but surely much effort will be dedicated to finding such one. For now, vincristine is and will remain a stable component of various anticancer regimens, the future of which will be linked mainly with tumor-targeting antibodies and further extending the drug combinations.

## Figures and Tables

**Table 1:** Ongoing (not yet recruiting; recruiting; enrolling by invitation; active, not recruiting) clinical trials with low-molecular-weight compounds combined with cyclophosphamide, doxorubicin, vincristine, and prednisone (May 2021).

Compound	Trade Name	Manufacturer	Molecular Target	Number of Clinical Trials	Reference
Etoposide	Vepesid^®^	Bristol-Myers Squibb, New York, NY, USA	Topoisomerase II	40	[33]
Lenalidomide	Revlimid^®^	Celgene corporation, Summit, CO, USA	Cereblon, Ikaros transcription factors	18	[34]
Ibrutinib	Imbruvica^®^	Pharmacyclics, Synnyvale, CA, USA	Bruton’s tyrosine kinase	4	[35]
Chidamide	Epidaza^®^	Schenzen Chipscreen, Shenzhen, China	Histone deacetylases	4	[36]
Nelarabine	Arranon^®^	GlaxoSmithKline, London, Great Britain	DNA synthesis	4	[37]
Azacitidine	Vidaza^®^	Bristol-Myers Squibb, New York, NY, USA		3	[38]
Decitabine	Dacogen^®^	Janssen Pharmaceuticals, Beerse, Belgium		3	[39]
Venetoclax	Venclexta^®^	Genentech, South San Francisco, CA, USA	B-cell lymphoma-2 protein	3	[40]
Dasatinib	Sprycel^®^	Bristol-Myers Squibb, New York, NY, USA	BCR-ABL ^1^ tyrosine kinase	3	[41]
Ponatinib	Iclusig^®^	ARIAD Pharmaceuticals, Cambridge, MA, USA		2	[42]
Romidepsin	Istodax^®^	Gloucester Pharmaceuticals, Cambridge, MA, USA	Histone deacetylases	2	[43]
Vorinostat	Zolinza^®^	Merck & Co., Kenilworth, IL, USA		2	[44]
Bortezomib	Velcade^®^	Takeda Pharmaceutical Company Limited, Tokyo, Japan	26S proteasome	2	[45]
Ixazomib	Ninlaro^®^		Proteasome subunit beta type-5	2	[46]
Acalabrutinib	Calquence^®^	AstraZeneca, Cambridge, Great Britain	Bruton’s tyrosine kinase	2	[47]
Zanubrutinib	Brukinsa^®^	BeiGene, Beijing, China		2	[48]
Umbralisib	Ukoniq^®^	Rhizen Pharmaceuticals AG, Basel, Switzerland	Phosphatidylinositol 3-kinase delta	2	[49]
Parsaclisib	n.a. ^2^	Innovent Biologics, Suzhou, China		1	[50]
Pralatrexate	Folotyn^®^	Allos Therapeutics, Westminster, CO, USA	Dihydrofolate reductase	1	[51]
Duvelisib	Copiktra^®^	Verastem Oncology, Needham, MA, USA	Phosphatidylinositol 3-kinase	1	[52]
Copansilib	Aliqopa^®^	Bayer, Leverkusen, Germany		1	[53]
Selinexor	n.a.	Karyopharm Therapeutics, Newton, MA, USA	Exportin-1	1	[54]
Iberdomide	n.a.	Celgene Corporation, Summit, CO, USA	Cereblon	1	[55]
TAK-659	n.a.	Takeda Pharmaceutical Company Limited, Tokyo, Japan	Spleen tyrosine kinase	1	[56]
Carfilzomib	Kyprolis^®^	Amgen, Thousand Oaks, CA, USA	20S proteasome	1	[57]

^1^ breakpoint cluster region-Abelson proto-oncogene; ^2^ a trading name not available.

**Table 2:** The number and status of all clinical trials currently evaluating vincristine on cancer patients (June 2021).

Status	Number of Clinical Trials	Reference
Not yet recruiting	30	[61]
Recruiting	178	[62]
Enrolling by invitation	1	[63]
Active, not recruiting	150	[64]

**Table 3:** Ongoing (not yet recruiting; recruiting; enrolling by invitation; active, not recruiting) clinical trials with monoclonal antibodies other than rituximab in combination with vincristine on patients suffering from various types of cancer (May 2021).

Monoclonal Antibody	Trade Name	Manufacturer	Molecular Target	Condition	Trial Identifier	Ref.
Tafasitamab	Monjuvi^®^	MorphoSys, Planegg, Germany	CD19 ^1^	Diffuse large B-cell lymphoma	NCT04824092	[125]
					NCT04661007	[126]
					NCT04134936	[127]
Atezolizumab	Tecentriq^®^	Roche, Basel, Switzerland	Programmed cell death-ligand ^1^	Solid tumors	NCT04796012	[148]
Nivolumab	Opdiva^®^	Bristol-Myers Squibb, New York, NY, USA		Primary mediastinal (thymic) large B-cell lymphoma	NCT04759586	[149]
				Peripheral T-cell lymphoma	NCT03586999	[150]
				Various lymphoma types	NCT03749018	[151]
					NCT03704714	[152]
Toripalimab	Tuoyi^®^	Junshi Biosciences, Shanghai, China			NCT04058470	[153]
Pembrolizumab	Keytruda^®^	Merck & Co., Kenilworth, IL, USA			NCT04058470	[153]
				Classical Hodgkin lymphoma	NCT03407144	[154]
Camrelizumab	AiRuiKa^®^	Jiangsu Hengrui Medicine, Lianyungang, China			NCT04113226	[155]
Durvalumab	Imfinzi^®^	MedImmune, Gaithersburg, MD, USA		Large B-cell lymphoma	NCT03003520	[156]
Sintilimab	Tyvyt^®^	Eli Lilly and Company, Indianapolis, IN, USA		Epstein-Barr virus-positive diffuse large B-cell lymphoma	NCT04181489	[157]
				Diffuse large B-cell lymphoma	NCT04023916	[158]
Avelumab	Bavencio^®^	EMD Serono, Rockland, USA; Pfizer, New York, NY, USA		Non-Hodgkin B-cell lymphoma	NCT03244176	[159]
Ublituximab	n.a. ^2^	LFB Group, Alès, France	CD20	Mantle cell lymphoma	NCT04692155	[116]
Blinatumomab	Blincyto^®^	Amgen, Thousand Oaks, CA, USA	CD19, CD3	B-cell acute lymphoblastic leukemia	NCT03518112	[128]
					NCT04448834	[129]
					NCT03914625	[130]
				Various leukemia types	NCT03147612	[131]
				B-cell acute lymphoblastic leukemia and lymphoma	NCT02877303	[132]
				Acute lymphoblastic leukemia	NCT03643276	[133]
					NCT02003222	[134]
Daratumumab	Darzalex^®^	Janssen Biotech, Horsham, PA, USA	CD38	Various types of lymphoma	NCT04139304	[139]
				Precursor cell lymphoblastic leukemia and lymphoma	NCT03384654	[140]
Isatuximab	Sarclisa^®^	Sanofi, Paris, France		Acute lymphoblastic leukemia, acute myeloid leukemia	NCT03860844	[141]
Inotuzumab	Besponza^®^	Pfizer, New York, NY, USA	CD22	Recurrent and refractory B-cell lymphoma and leukemia	NCT03991884	[162]
					NCT03851081	[163]
					NCT02981628	[164]
				Acute lymphoblastic leukemia	NCT03249870	[165]
				B-cell acute lymphoblastic leukemia and lymphoma	NCT02877303	[132]
				Various leukemia types	NCT01925131	[166]
				Lymphoblastic leukemia	NCT04747912	[167]
					NCT04307576	[168]
				B-acute lymphoblastic leukemia	NCT03150693	[169]
					NCT01371630	[170]
Obinutuzumab	Gazyva^®^	Genentech, South San Francisco, CA, USA	CD20	Advanced follicular lymphoma	NCT03817853	[171]
				B-cell lymphoma, non-Hodgkin lymphoma	NCT03467373	[118]
				Follicular lymphoma	NCT03269669	[172]
				Lymphoma	NCT02529852	[173]
				Non-Hodgkin lymphoma	NCT01332968	[174]
Dinutuximab	Unituxin^®^	United Therapeutics, Silver Spring, MD, USA	Ganglioside G2	Ganglioneuroblastoma, high risk neuroblastoma	NCT03786783	[160]
Mosunetuzumab	-	Genentech, South San Francisco, CA, USA	CD20, CD3	B-cell non-Hodgkin lymphoma	NCT03677141	[117]
Polatuzumab	Polivy^®^		CD79B	Various lymphoma types	NCT04231877	[145]
				Diffuse large B-cell lymphoma	NCT03274492	[146]
Brentuximab	Adcetris^®^	Takeda Oncology, Cambridge, CA, USA	CD30		NCT02734771	[135]
				Hodgkin lymphoma	NCT02398240	[136]
					NCT02166463	[137]
					NCT01920932	[138]
Ganitumab	-	Amgen, Thousand Oaks, CA, USA	Type 1 insulin-like growth factor receptor	Solid tumors	NCT02306161	[161]
Ofatumumab	Arzerra^®^	Novartis, Basel, Switzerland	CD20	Mantle cell lymphoma	NCT01527149	[119]
Glofitamab	-	Genentech, South San Francisco, CA, USA		Diffuse large B-cell lymphoma	NCT03467373	[118]
Siplizumab	-	BioInvent, Lund, Sweden	CD2	T-cell lymphoma	NCT01445535	[121]
Alemtuzumab	Campath^®^	Sanofi, Paris, France	CD52	Hodgkin lymphoma, diffuse large B-cell lymphoma	NCT01030900	[142]
				T-cell lymphoma	NCT00069238	[143]
				Acute lymphoblastic leukemia	NCT01256398	[144]

^1^ cluster of differentiation; ^2^ a trading name not available.

## Data Availability

Not applicable.

## Abbreviations

A549	Adenocarcinomic human alveolar basal epithelial cells
ABL	Tyrosine-protein kinase transforming protein Abl
ABCB1/Flp-In^™^-293	Fetal kidney cell line stably expressing P-gp
ABVD	Chemotherapy with doxorubicin, bleomycin, vinblastine, and dacarbazine
BCR	Breakpoint cluster region protein (renal carcinoma antigen NY-REN-26)
BEACOPP	Chemotherapy combination of bleomycin, etoposide, doxorubicin, cyclophosphamide, vincristine, procarbazine, prednisolone
CHOP	Chemotherapy with cyclophosphamide, doxorubicin, vincristine, and prednisone
CNS	Central nervous system
CVD	Chemotherapy with cyclophosphamide, vincristine, and dacarbazine
DNA	Deoxyribonucleic acid
Eca109/VCR	Human cells from esophageal cancer
EPOCH-(R)	Combination therapy with cyclophosphamide, doxorubicin, vincristine, prednisone, rituximab, and etoposide
EMA	Chemotherapy combination of methotrexate, etoposide, and dactinomy-cin
EMA/CO	Chemotherapy combination of etoposide, methotrexate, actinomycin D, cyclophosphamide, and vincristine
FDA	US Food and Drug Administration
G401	Human cells from a rhabdoid tumor of the kidney
IC_50_	Half-maximal inhibitory concentration
*IDH1/2*	Gene encoding nicotinamide adenine dinucleotide phosphate-dependent isocitrate dehydrogenases
IL-1	Interleukin 1 receptor
KB-V	Cervical adenocarcinoma cell line
LGG	Low-grade gliomas
LC3-I	Microtubule-associated proteins 1A/1B light chain 3B
lncRNA	Long non-coding RNA
MCF-7/ADR	Multidrug-resistant breast cancer cell line
*MEG3*	Maternally expressed gene 3
MOPP	Combination of vincristine, procarbazine, prednisone, and mechlorethamine
NHP2L1	Non-histone protein 2-like protein 1
*NOXA*	Gene coding phorbol-12-myristate-13-acetate-induced protein 1
PCNSL	Primary central nervous system lymphoma
PCV	Chemotherapy combination of procarbazine, lomustine, and vincristine
P-gp	P-glycoprotein
*PMAIP1*	Gene encoding phorbol-12-myristate-13-acetate-induced protein 1
R-CHOP	Combination therapy with cyclophosphamide, doxorubicin, vincristine, prednisone, and rituximab
R-CHOPE	Combination therapy with cyclophosphamide, doxorubicin, vincristine, prednisone, rituximab, and etoposide
RNA	Ribonucleic acid
VIPN	Vincristine-induced peripheral neuropathy
WERI-Rb-1	Human cells from retinoblastoma
XIST	X-inactive specific transcript
Y79	Human cells from retinoblastoma
